# Supplementary material for: DACCOR–Detection, characterization, and reconstruction of repetitive regions in bacterial genomes
Source: PeerJ. 2018 May 29;6:e4742. doi: 10.7717/peerj.4742 (PMC5983011; doi:10.7717/peerj.4742)
Supplement: Supplemental Information 1 [file peerj-06-4742-s001.pdf]

Repeat	R_{A_1}				R_{B_1}						R_{A_2}				R_{B_2}				
Sequence	A	C	T	G	T	T	G	A	G	G	A	C	T	C	T	T	G	A	A
Position	0	1	2	3	4	5	6	7	8	9	10	11	12	13	14	15	16	17	18

Step 3:
repeats without mismatches

Repeat sequences	start positions
ACT	0,10
TTGA	4,14

Step 4:
add mismatch marker ’0’

Repeat sequences	start positions
ACT0	0,10
TTGA0	4,14

Repeat	R'_{A_1}				R'_{B_1}						R'_{A_2}				R'_{B_2}				
Sequence	A	C	T	G	T	T	G	A	G	G	A	C	T	C	T	T	G	A	A
Position	0	1	2	3	4	5	6	7	8	9	10	11	12	13	14	15	16	17	18

Step 5:
resolve regions with mismatches
$4 - 2 + 1 = 14 - 12 + 1 \leq m$ (here $m = 1$)

Repeat sequences	start positions
ACT0	0,10
TTGA0	4,14
ACT0TTGA0	0,10

Step 6:
Filtering, removing trailing ’0’s,
and replacment of ’0’s by ’N’s

One repetitive region: ACTNTTGA
