# Supplementary figures and images for: DACCOR–Detection, characterization, and reconstruction of repetitive regions in bacterial genomes
Source: PeerJ. 2018 May 29;6:e4742. doi: 10.7717/peerj.4742 (PMC5983011; doi:10.7717/peerj.4742)

# Runtime comparison between DACCOR and Vmatch

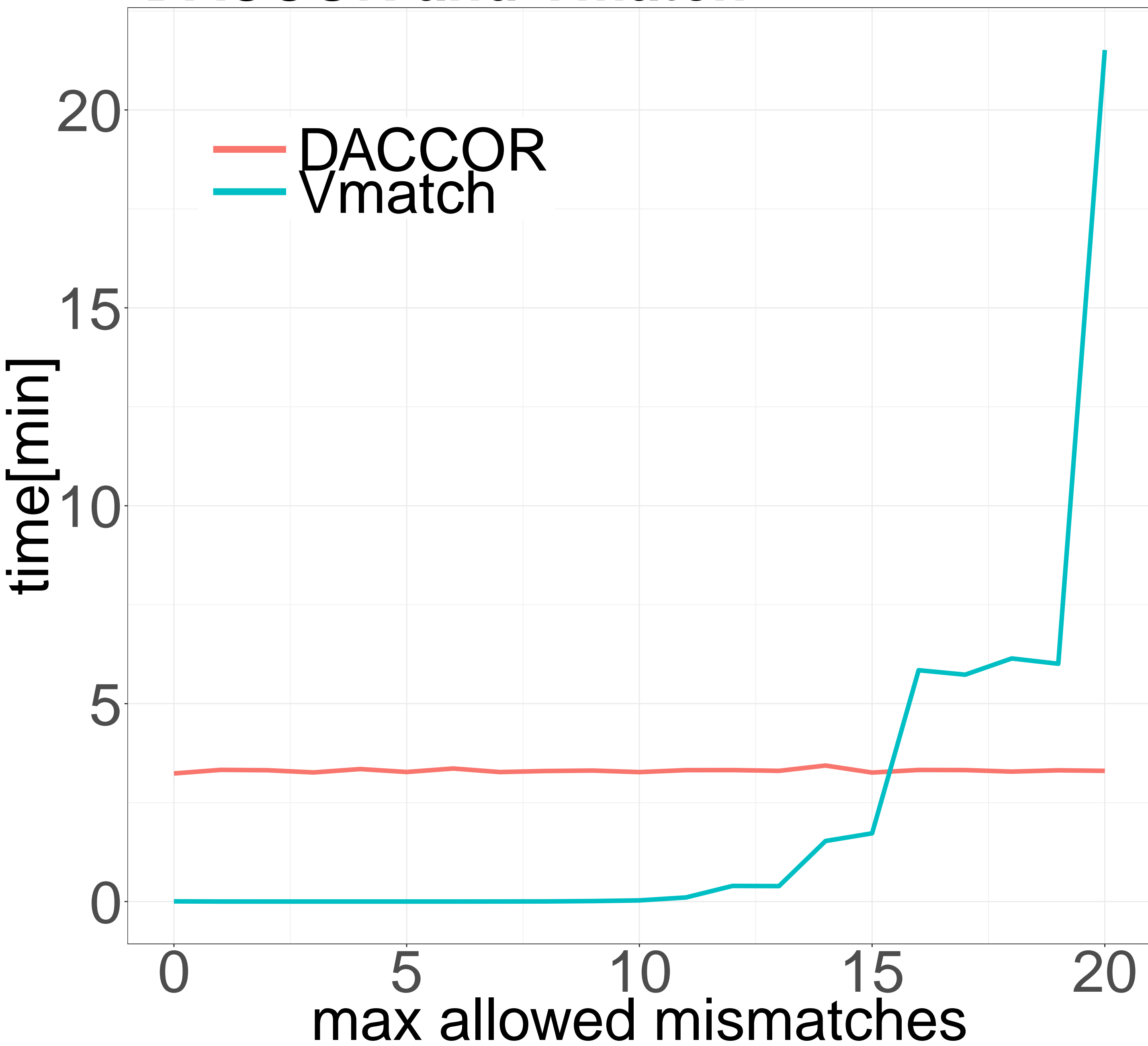

Supplement: Supplemental Information 2 [file peerj-06-4742-s002.pdf]

# *Runtimes on the Nichols genome*

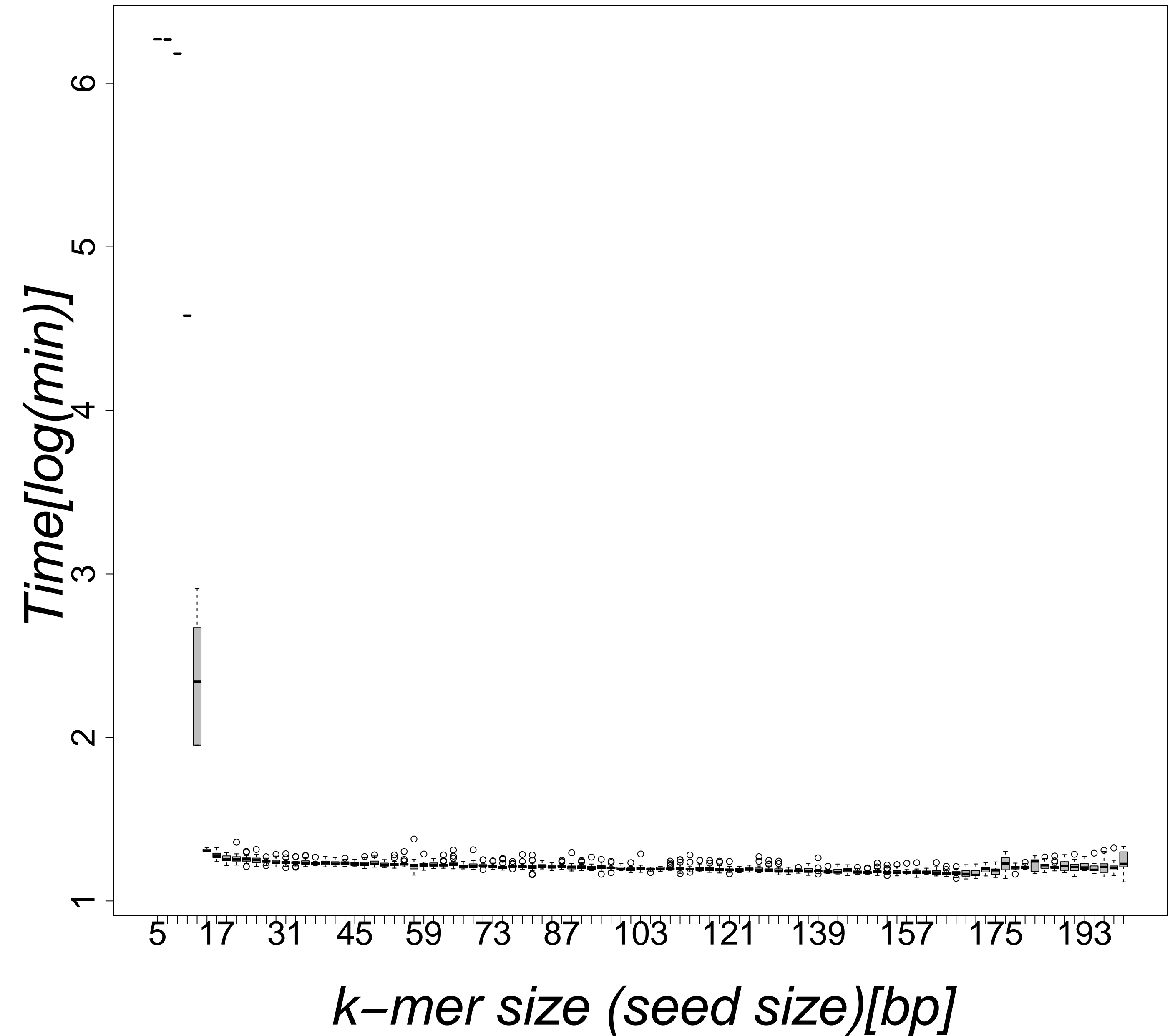

Supplement: Supplemental Information 3 [file peerj-06-4742-s003.pdf]

sample names

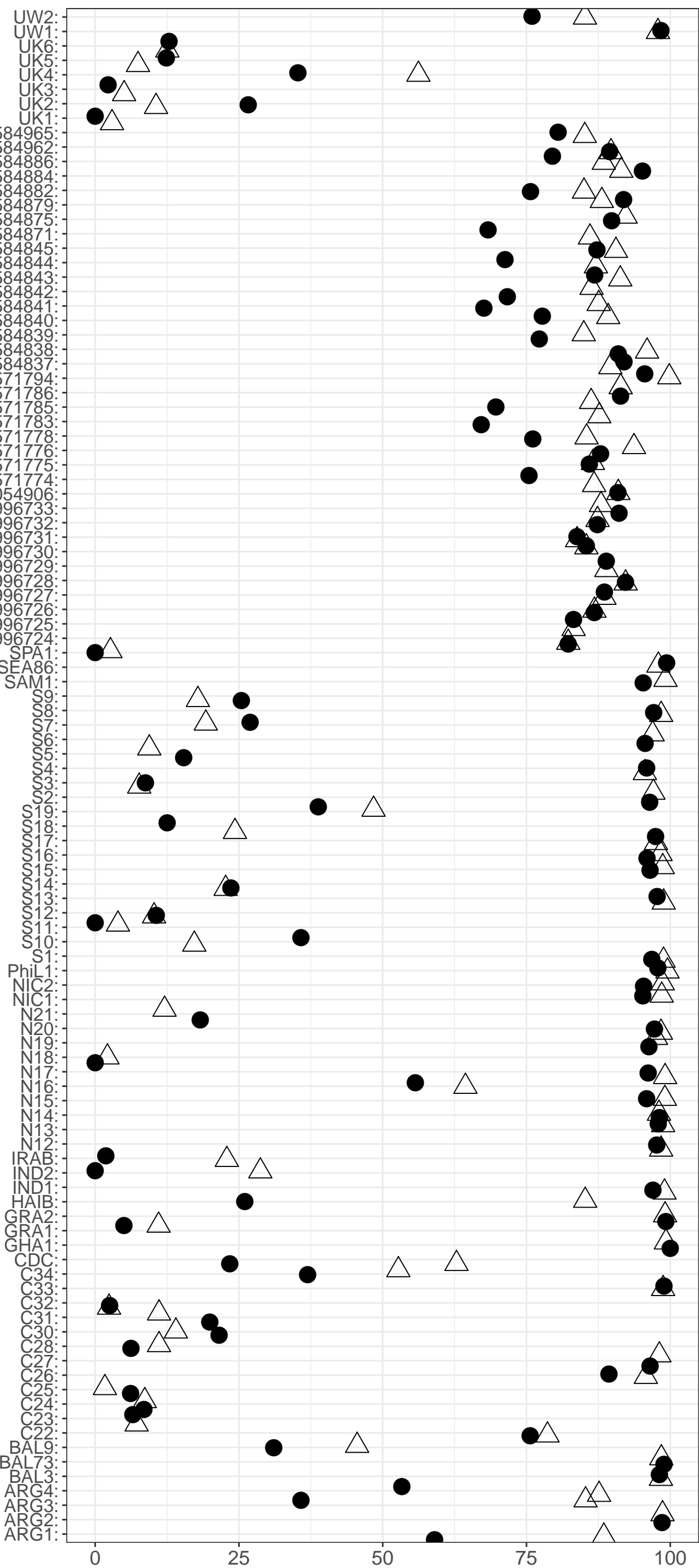

rRNA  
● 16S  
△ 23S

rel. difference (%)

Supplement: Supplemental Information 4 [file peerj-06-4742-s004.pdf]
